# Ultrathin Fluidic Laminates for Large‐Area Façade Integration and Smart Windows

**DOI:** 10.1002/advs.201600362

**Published:** 2016-11-21

**Authors:** Benjamin P. V. Heiz, Zhiwen Pan, Gerhard Lautenschläger, Christin Sirtl, Matthias Kraus, Lothar Wondraczek

**Affiliations:** ^1^ Otto Schott Institute of Materials ResearchUniversity of JenaFraunhoferstrasse 607743JenaGermany; ^2^ Center for Energy and Environmental Chemistry – CEECUniversity of JenaPhilosophenweg 707743JenaGermany; ^3^ SCHOTT Technical Glass Solutions GmbHOtto‐Schott‐Strasse 1307745JenaGermany; ^4^ Chair of Steel and Hybrid StructuresUniversity of WeimarMarienstraße 13D99423WeimarGermany

**Keywords:** energy efficient buildings, energy harvesting, fluidic window, glass, smart window, solar energy

## Abstract

Buildings represent more than 40% of Europe's energy demands and about one third of its CO_2_ emissions. Energy efficient buildings and, in particular, building skins have therefore been among the key priorities of international research agendas. Here, glass–glass fluidic devices are presented for large‐area integration with adaptive façades and smart windows. These devices enable harnessing and dedicated control of various liquids for added functionality in the building envelope. Combining a microstructured glass pane, a thin cover sheet with tailored mechanical performance, and a liquid for heat storage and transport, a flat‐panel laminate is generated with thickness adapted to a single glass sheet in conventional windows. Such multimaterial devices can be integrated with state‐of‐the‐art window glazings or façades to harvest and distribute thermal as well as solar energy by wrapping buildings into a fluidic layer. High visual transparency is achieved through adjusting the optical properties of the employed liquid. Also secondary functionality, such as chromatic windows, polychromatism, or adaptive energy uptake can be generated on part of the liquid.

## 1 Introduction

Glass has become an essential component in modern building skins. This is primarily due to its visual transparency, but also due to its surface quality and general attractiveness, its mechanical properties and its long‐term stability in a wide range of climatic and solar irradiance conditions. More recently, glasses have also seen significantly increasing popularity as a structural material, what resulted in a rising number of applications and steadily evolving material demands. The addition of new functionality to classical glass panes has been in the focus of intense research efforts, emerging into the so‐called smart‐window sector with significant expectations for the near and mid‐term future. Some concrete examples involve tailored shading or lighting,^1^, ^2^, ^3^, ^4^ passive and active shielding from noise^5^ or electromagnetic radiation, integration of transparent electronics such as radio‐frequency antennas,^6^ displays or light‐emitting devices,^7^ solar cells,^8^, ^9^, ^10^ polychromatism or the control of light emission,^11^, ^12^, ^13^ both for solar shielding and reduction of thermal losses from within the building.

However, the key requirements of windows and, to often similar extent, façade systems remain visual appearance and thermal performance. According to several recent studies, buildings account for more than 40% of the total European energy consumption^14^ and generate more than one third of the CO_2_ emissions. In order to achieve the 2050 agenda^15^ of at least 80% reduction in CO_2_ emissions compared to 1990, and a parallel cut in energy demand by 50%, significant material improvements in the building skin and, in particular, in windows are required.^16^ Clearly, this also applies across other parts of the world.^17^

Several technologies are being investigated and further innovated toward this goal. Among these are multiple (quadruple) glazings^18^ and low‐emission coatings,^19^, ^20^, ^21^, ^22^ laminate glazings which incorporate solar‐thermal collectors,^23^ luminescent concentrators,^24^ latent heat storage,^25^ ultrathin glass membranes, or light‐guiding micromirrors,^26^ but also switchable windows (chromogenics^1^, ^2^, ^13^, ^27^, ^28^, ^29^, ^30^, ^31^, ^32^, ^33^, ^34^, ^35^, ^36^, ^37^, ^38^), window‐integrated polymer‐dispersed liquid crystals,^1^, ^2^, ^39^, ^40^, ^41^, ^42^ and suspended particle devices.^1^, ^2^, ^43^

Among the concepts of adaptive building skins has been the idea of wrapping a building into a layer of a liquid, whereby this liquid could act as a tunable cooling (or heating) reservoir. In the most literal way, this has been demonstrated on the islandic pavilion at the world exposition in Hannover, Germany, in 2000.^44^ Parallel and subsequent efforts to harness liquids within façade elements have been restricted to filling the cavity between two sheets of glass in a double‐ or multiglazing device (e.g., for algae reactors^45^) or, for example, to thin layers of immobilized liquids such as the use of liquid crystal layers for glare/daylight control.^1^ Similarly, also at least occasionally liquid phase change materials have been considered as a filling material for energy storage.^46^ While the former approach has mostly failed due to issues such as mechanical stability, internal fouling, or pumping and ancillary maintenance, the latter approach is, per se, not useful for liquid circulation and quantitative energetic throughput. Yet another option might hence be sought on the distinct area of microfluidics. Here, micropumping^47^ and microfluidic cooling^48^ have seen significant technological progress, but system size, throughput, and associated cost seem to require a change of paradigm before transfer to large‐scale application in façades and windows might become possible.

**Figure 1:**
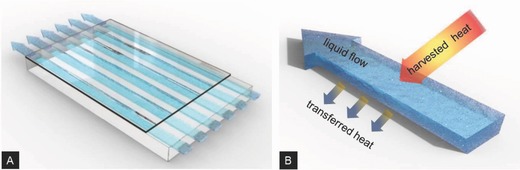
Fluidic device for large‐area window and façade integration. A) Laminate system of microchannel glass pane and thin‐sheet cover with the functional liquid flowing through the channels. B) Schematic of the exchange of ambient heat and solar energy across an individual channel.

**Figure 2:**
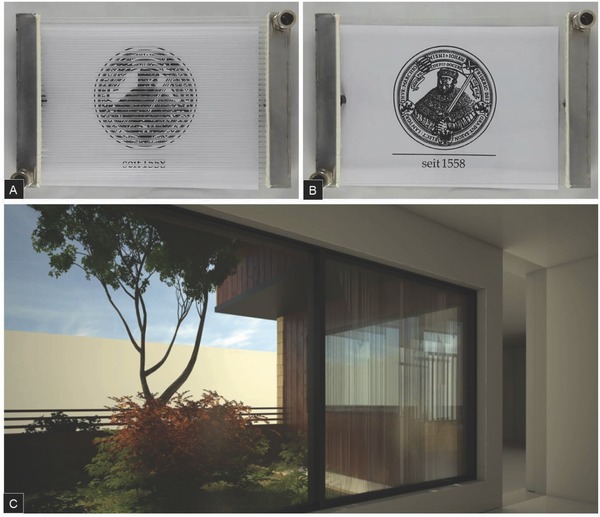
Visual appearance of a microfluidic device for window integration. A,B) 30 × 21 cm^2^ demonstrator without liquid (empty channel array – left) and filled with a liquid with refractive index matched to that of the capillary glass (right), respectively. C) Computational rendering of the interior view through a window system according to Figure 1a (see text for details).

In this report, we introduce glass–glass fluidic devices for large‐area integration with adaptive façades and smart windows. We show that these enable harnessing and dedicated control of liquid functionality in a large‐area window system. In the present example, a fluid is used as a reservoir and transport system to harvest external heat as well as solar energy, and to distribute this energy within the building. As shown in **Figure** **1**, this combines a microchannel glass pane and a thin cover sheet with tailored mechanical performance. The functional liquid is flowing through these microchannels. The thin‐sheet surface is subsequently exposed to the reservoir from which heat is to be harvested (or to which heat is to be delivered). Depending on application, this can be the exterior or interior side of a window. For integration with present building technologies, the thickness of the overall device is adapted to the thickness of a single glass sheet in conventional glazings. Experimental demonstration and computational verification of this concept are provided. Through adjusting the optical properties of the liquid, the visual appearance of the overall system can be tailored between fully transparent and various degrees of light refraction or scattering (Figure 1). This leads to a wide range of potential applications, and a broad variety of façade constructions. Toward this end, as shown in **Figure** **2**C, the visual appearance of the reported devices in real‐world façades is also considered.

## 2 Results and Discussion

### 2.1 General

Manufacture of capillary glass sheet from a low‐expansion borosilicate with a capillary cross‐section of about 3 mm^2^ and an intercapillary distance of about 3 mm by milling did not cause any mechanical failure. The transition between the capillary ground and the flight flank does not form a sharp edge, and minor flaking occurred during the milling process. However, both defects did not have relevant consequences for the stability of the system, in particular, at a residual glass thickness of 4 mm. The inner channel roughness of 2.96 ± 0.29 µm (obtained from scanning profilometry) was assumed to not have significant effects on the flow behavior inside the capillaries and was neglected in the computational verifications. As will be discussed later, the good match between experiment and calculation confirmed that this simplification does not have measurable consequences for the present study. While not performing mechanical testing at this point, further stabilization is expected from the contribution of the cover glass sheet. Bonding of the cover glass through UV curing is strongly facilitated by the specific UV transparency of the employed type of glass. No defects such as leakage or bond heterogeneity could be observed throughout the experiments. They are hence supposed to have no determining influence on the present investigations and were thus neglected for the simulation model.

### 2.2 Thermal Properties and Heat‐Exchange

**Figure 3:**
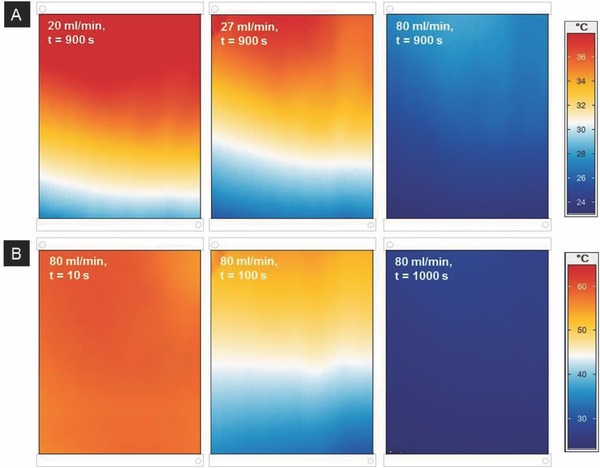
Infrared imaging of fluidic window elements. Images are shown for a cooling experiment from about 60 °C (steady heat injection of 510 W m^−2^) with constant inlet temperature of 23 °C. A) Effect of flow rate after achieving steady state. B) Effect of time at a flow rate of 80 mL min^−1^. In all images, the input is on the bottom right and the output on the top left.

**Figure 4:**
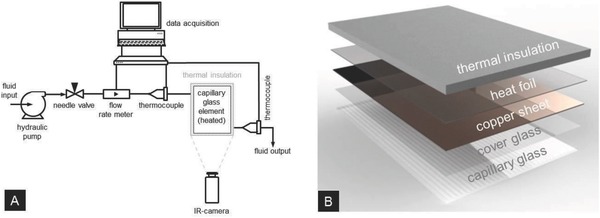
Schematic of the experimental set‐up for thermal performance testing on fluidic window elements. A) General set‐up. B) Setting‐up of the capillary glass element.

**Figure** **3**A shows changes in surface temperature of fluidic window elements as obtained from an infrared camera as a function of the flow rate for a heat transfer experiment with constant heat injection according to **Figure** **4**. Flow rates around 20–30 mL min^−1^ only have a relatively small effect on the overall window temperature, meaning that the injected heat is not efficiently harvested through the liquid. Uniform cooling of the entire window is obtained for higher flow rates, e.g., 80 mL min^−1^. Practically regardless of the flow rate, a steady‐state is reached within about 15 min for all investigated flow rates. As an example, this is shown for a flow rate of 80 mL min^−1^ in Figure 3B. A nearly homogeneous temperature profile, ranging from 23 to 28 °C, is achieved after a time of 1000 s. Then, the present system is providing an output of about 30 W of thermal power.

**Figure 5:**
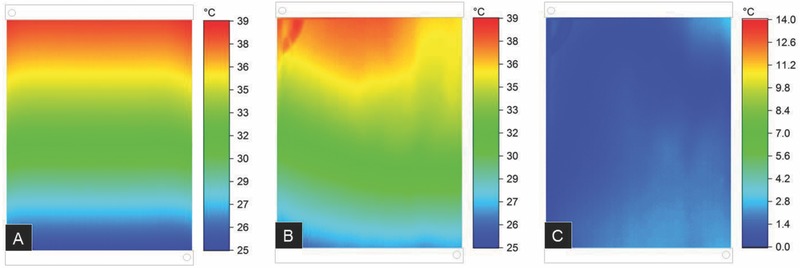
FEM computational verification of steady‐state heat exchange across fluidic windows. Images are temperature distributions across a 300 × 210 mm^2^ fluidic window with heat injection of 510 W m^−2^ for an inlet temperature of 23 °C and a flow rate of 27 mL min^−1^. A) FEM simulation. B) Experimental data. C) Absolute difference between experimental and computational data.

These experimental observations are well‐reproduced by computational data. In order to test the accuracy of the simulation model, we compared the experimental infrared images to computer‐generated patterns. In **Figure** **5**A,B, this is shown for a flow rate of 27 mL min^−1^ after achieving a steady state. Figure 5C presents the difference image between experimental and computational data. Across the whole window area, a maximum deviation of 2.2 °C and a standard deviation of 1.0 °C were observed for these experimental conditions. At the outlet, a temperature difference of only 0.4 °C is observed (for an overall temperature difference of 15 °C between inlet and outlet). The main deviations occur in the edge regions, and close to inlet and outlet, respectively, due to heat transfer and isolation issues at the edges which were not taken into account in the simulation, and, eventually, slight heterogeneities in the flow between individual capillaries. Deviations become even smaller for higher flow rates, i.e., when a more homogeneous temperature distribution is achieved experimentally, and for larger plate sizes. This good match between experiment and simulation data verifies the assumptions regarding the use of the present concept for efficient heat harvesting in fluidic windows. It also enables rapid optimization through computational simulation.

**Figure 6:**
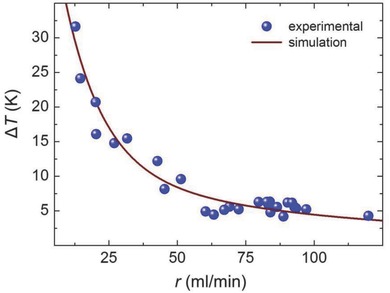
Thermal efficiency of microfluidic window. The graph shows the temperature difference Δ*T* between inlet and outlet of the microfluidic reactor for different flow rates *r*. Data are shown for a unit size of 300 × 210 mm^2^.

In **Figure** **6**, the temperature difference Δ*T* between inlet and outlet is depicted for varying flow rates, again comparing experimental data and simulation. Both datasets show a very good match. For practical purpose, neither very high nor very low flow rates might be desirable. Hence, an as‐low‐as‐possible flow rate can be chosen as a trade‐off so as to achieve a homogeneous temperature profile (low Δ*T*).

**Figure 7:**
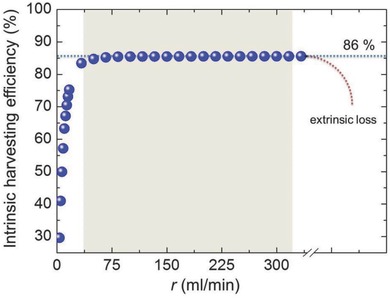
Intrinsic harvesting efficiency. The graph shows the ratio between injected and transported heat as a function of flow rate *r*, obtained with the present prototype design. At flow rates above ≈300 mL min^−1^, extrinsic loss such as caused by pumping efficiency start to dominate the overall efficiency. Data are shown for a unit size of 300 × 210 mm^2^.

The above observations enable predictions based on the system's thermal behavior. The intrinsic efficiency of the system represents the ratio between the amount of energy transferred to the system and the amount of energy which is effectively absorbed by the fluid. By assuming that the simulation model is valid, the system's intrinsic efficiency can be predicted. **Figure** **7** provides the predicted intrinsic efficiency for flow rates ranging from 0 to 330 mL min^−1^, assuming a pump efficiency of 100%. According to this, no further intrinsic improvement can be achieved for flow rates higher than about 50 mL min^−1^. Notwithstanding, this does not take into account extrinsic parameters such as the pumping efficiency and, thus, the global efficiency. For example, the global efficiency will be reduced when further increasing the flow rate after passing an optimum value due to nonlinearly increasing pumping losses. In order to predict the extrinsic efficiency and thus the global efficiency of the system, a complete life‐cycle‐analysis of the system will thus have to be carried out.

### 2.3 Fluid Motion within the Microchannels

**Figure 8:**
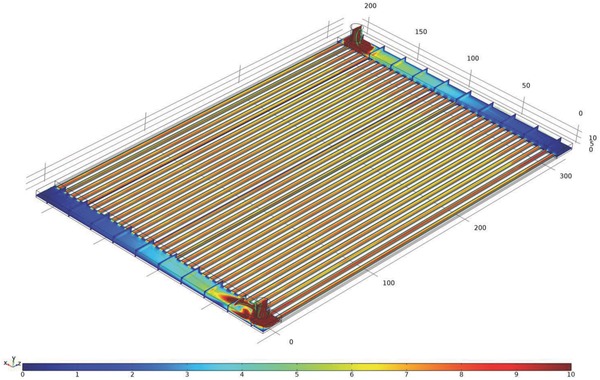
Flow velocity distribution within the microfluidic device. Regions with a velocity magnitude higher or equal to 10 mm s^−1^ are colored in dark red.

Guaranteeing homogenous flow within the microchannels avoids potential particle accumulations in some parts of the reactor, prevents energy losses through uniform heat dissipation and guarantees an even liquid and (in case the liquid is intentionally loaded with particles) particle distribution in the façade element, for example, in an application as a shading device. Therefore, it is important to achieve a homogeneous distribution of the fluid over all capillaries. However, the first two thermal images in Figure 3A suggest that for low flow velocities (around 20–27 mL min^−1^), the fluid distribution is slightly inhomogeneous. Close to the fluid inlet, the velocity seems to be lower than at the opposite corner. By increasing the input flow rate, this trend decreases and finally disappears, leading to a homogeneous distribution of the fluid over all capillaries, as suggested in Figure 3B. Regarding the fluid motion within the system, the simulation model could not be validated experimentally, yet. Indeed, according to Figure 5, the calculated flow velocity distribution within the system does not perfectly match reality. According to the simulation results, a homogeneous distribution would rather be expected (see **Figure** **8**).

### 2.4 Optical Properties and Rendering

**Figure 9:**
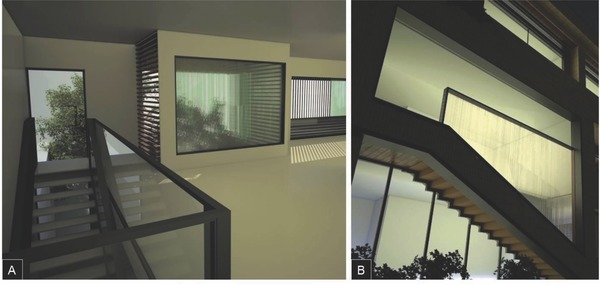
Computational rendering of an experimental façade implementing fluidic window devices as reported here. A) Indoor by day. B) Outdoor by night (see text for details).

In the last years, about half of all big office and administration buildings (with height >100 m) as well as a multitude of smaller buildings were designed in partially or fully transparent multifunctional design. The main reason for the increased use of multifunctional façades or windows is their capability to contribute to sustainability in terms of resources/energy saving and to an improvement of comfort, subject to the previous paragraphs. A further issue is the visual appearance and, associated, the contribution to a pleasant indoor climate. The façade has to provide sufficient transparency and still secure privacy, what is a major boundary rule in the designing of façades. While the fluidic windows presented here draw on the ideal transparency of nonstructured, homogeneous glasses, they enable some new design features which have previously not been available. As shown in Figure 2C, depending on the optical properties of the liquid and the shape of the channel cross‐section, translucency, and shading effects occur. These can dedicatedly be tailored (**Figure** **9**A). Besides indoor appearance, also outdoor appearance can be a major design target (Figure 9B).

## 3 Conclusions

In summary, we presented glass–glass fluidic devices for large‐area integration with adaptive façades and smart windows. These devices comprise a heat‐storing fluid which is transported through microchannels. The latter are directly integrated into the glass pane and, hence, enable very thin structure design for integration with state‐of‐the‐art glazing, low weight, and appealing optical properties. High mechanical reliability is primarily generated by the thin cover glass, which can be manufactured from various types of standard or super‐strong glass sheet, depending on target application and the potential wish for secondary toughening. Here, we generated a flat‐panel laminate with thickness adapted to a single glass sheet in conventional windows for the purpose of harvesting and distributing thermal as well as solar energy. High visual transparency is achieved through adjusting the optical properties of the employed liquid. Also secondary functionality such as chromatic windows, polychromatism or adaptive energy uptake can be generated on part of the liquid.

As a prerequisite for further optimization, the influence of the flow rate on the system's thermal properties and fluid dynamics have been investigated. Experimental studies have shown that lower flow rates generate higher temperature differences across the window, but also a less homogeneous flow distribution over the capillaries. Furthermore, a maximum limit for the system's intrinsic efficiency has been predicted from simulation results, thus suggesting an upper limit for the volumetric flow rate. According to the performed studies, a suitable range for the volumetric flow rate lies between 40 and 80 mL min^−1^ for harvesting an injected thermal load of about 500 W m^−2^. Future low‐cost large‐area microchannel plates can be manufactured trough rolling. This will enable a novel component in the design of ultraefficient and adaptive building skins toward low‐carbon and negative‐energy buildings.

## 4 Experimental Section

**Figure 10:**
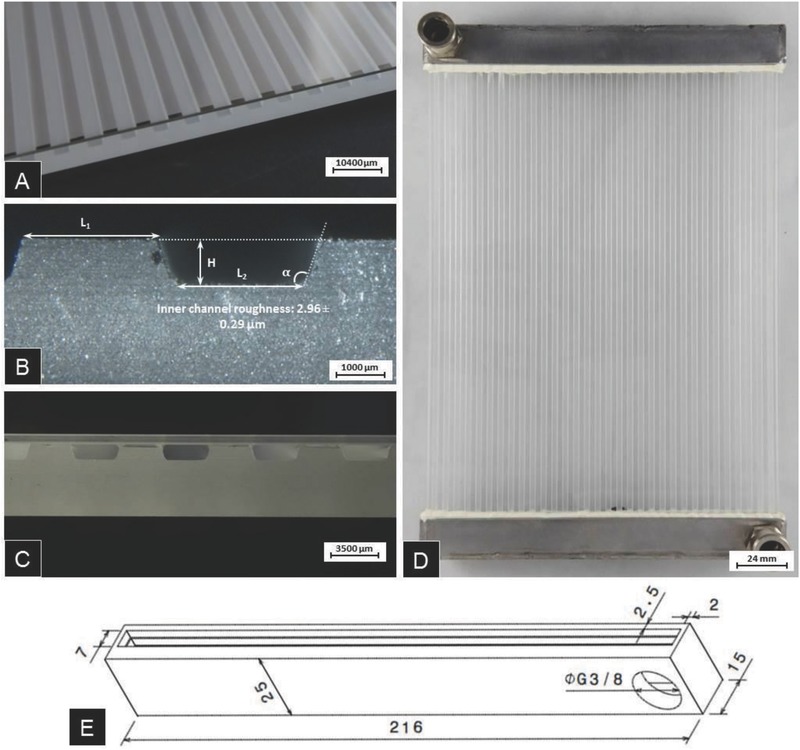
Experimental realization of a glass–glass microfluidic device. A) Overview photograph. B) Cross‐section of as‐machined channel. C) Cross‐section of bonded microchannel pane and cover sheet. D) Laminate with duct and distributer. E) Geometry of steel connector (labels: mm).

*Capillary Glass Element*: The principle design of the present device is shown in Figure 1a. For demonstration purposes, here, a device size of 300 × 210 mm^2^ was focused. As laminate material, a borosilicate float glass was chosen (Borofloat 33, Schott TGS), providing high surface quality, high transparency, and high mechanical stability.^49^ Relevant properties of this glass are its transition temperature *T*_g_ of 525 °C, a coefficient of linear thermal expansion α_(20–300 °C)_ of 3.25 × 10^−6^ K^−1^, and a thermal conductivity λ_(90 °C)_ of 1.2 W m^−1^ K^−1^.^50^ Its UV absorption edge lies at 275 nm. We used a 5 mm glass sheet into which an array of 32 trapezoidal channels was milled, and a 0.7 mm cover glass sheet (**Figure** **10**). Both glasses were bonded together with a robot‐printed UV‐curing acrylate (Delo‐Photobond GB368, DELO Industrial Adhesives), whereby bonding was facilitated through the high UV transparency of the employed type of glass. The selected adhesive was applied at a feed rate of 5 mm s^−1^ and a pressure of 5 bar by means of a *xy*‐displaceable dispenser unit (Fisnar 7400C, I&J Fisnar, USA) equipped with a dosing syringe with a needle diameter of 0.25 mm. Curing was performed with UV light (Delolux LED curing lamp, DELO Industrial Adhesives) at a wavelength of 400 nm. The device was exposed during 120 s at an UV‐A intensity of 100 mW cm^−2^. The employed adhesive is optically transparent across the visible spectral range with a refractive index of 1.506.^51^ Its Young's modulus is 900 MPa and the thermal expansion coefficient in the temperature range of 25–140 °C is 236 ppm K^−1^.^51^ The cured adhesive has a high temperature resistance, reportedly across the temperature range from −40 to +120 °C.^51^

The cross‐section of an as‐machined capillary is shown in Figure 10B, and of the bonded system in Figure 10C. The characteristic dimensions of the capillaries as determined by optical microscopy after machining are given in **Table** **1**. The cappillary glass element was fit to a distributer and duct machined from stainless steel (Figure 10D–E). Here, connector, distributer, inlet, and outlet geometry were designed according to finite element simulation feedback for generating homogeneous flow within all channels. The stainless steel distribution channel comprised a rectangular cross‐section of 93.5 mm^2^ and a length of 210 mm, connecting all capillaries. Equivalent inlet and outlet diameters of 10 mm were chosen. Fitting was done with a polyurethane adhesive (Delo‐Pur 9895, DELO Industrial Adhesives) which was pressurized into the 650 µm cavity between glass and steel structures to allow for some variations in thermal expansion.

**Table 1:** Characteristic dimensions of the capillaries after machining

Land width *L*_1_ [µm]	Capillary width *L*_2_ [µm]	Capillary depth *H* [µm]	Angle ground‐flight flank α [°]
2957 ± 20	2924 ± 30	961 ± 11	106 ± 1

*Testing*: The devices described above were employed for experimentally testing their heat‐exchange properties as fluidic windows. A schematic of the testing facility is shown in Figure 4.

In the reported testing situation, deionized water was used as the heat‐exchanger liquid (in the photograph of Figure 2c, paraffin oil with a refractive index of 1.462–1.472 is employed). Relevant liquid data as also used in the subsequent computational verifications are from the Multiphysics Simulations Material Library included in the simulation software. In a typical experiment, water at a temperature of 23 °C was pumped into the system with a hydraulic pump at a pressure of 3.1 × 10^6^ Pa and a flow rate of 10–125 mL min^−1^ (regulated through a microvalve at the system inlet). Within this range, flow remains fully laminar. For every measurement, time, flow rate, and temperature were determined in line with an automatic acquisition routine.

Controlled areal heat injection was performed on the cover‐side of the capillary element, using a copper plate contacted with a heat transfer paste (Amasan T12, Jürgen Armack GmbH, Germany) to the cover glass, and heating homogeneously across the whole area with electric heating foils. In this way, the injected heat can be calculated more accurately as compared to, e.g., when using a solar simulator. The voltage at the heater was controlled through a DC supply unit. The supplied power was then calculated using the measured voltage and current. In the presented case, the power was 510 W m^−2^. In order to prevent heat losses and ensure that all the thermal energy is effectively transferred to the glass element, the whole system, except for the backside of the microstructured pane, was thermally isolated using Styrofoam. A microbolometer infrared camera (VarioCAM HD, InfraTec, Germany) was used to record the temperature distribution on the system's backside (side of microstructured glass pane) within the wavelength range of 7.5–14 µm. In this spectral range, the employed glass is intransparent, thus, the surface temperature is recorded. All measurements were conducted as a function of flow rate and inlet temperature, for heating as well as for cooling. In a typical cooling experiment, prior to initiating fluid flow, the glass element was heated to an initial temperature ranging between 55 and 60 °C. Water with an inlet temperature of 23 °C was then pumped through the channels while maintaining heat injection.

*Computational Verification*: To complement the experimental work, a 3D finite element model (FEM) was developed on the FEM software platform COMSOL Multiphysics v.5.1 to determine the steady‐state heat and flow distribution in the aforementioned device. The temperature dependency of the fluid's properties, such as the heat capacity, the coefficient of cubic expansion, the relative heat transfer, the relative pressure drop, the fluid mass density, the dynamic viscosity, and the thermal conductivity were taken into account in this model.

**Figure 11:**
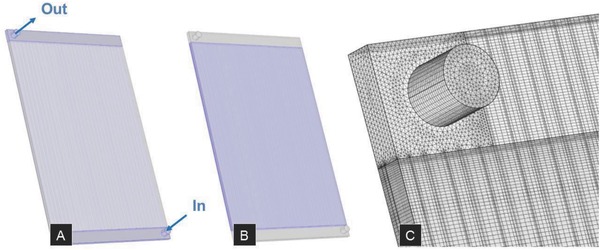
FEM‐model for computational verification. A) Fluid body. B) Glass body. C) Zoom at the inlet region of the fluid model, visualizing discretization.

Regarding the boundary conditions, a fully developed, steady‐state laminar flow in a 3D model with non‐slip‐boundary conditions was considered. The fluid was treated as being incompressible and Newtonian, and gravity was taken into account as body force. Further, to guarantee high calculation accuracy, a fine discretization mesh was chosen, discretizing the model in about 7.9 million tetrahedral and hexahedral elements (see **Figure** **11**).

In this way, both the flow velocity distribution within the channels and the system's global thermal behavior were predicted as specified below.

*Fluid Motion*: In order to describe the motion of the viscous fluid substances within the system, the Navier–Stokes Equation (1) was applied

(1) $$\rho \left(\frac{\partial v}{\partial t}+\left(v\cdot \nabla \right)v\right)=f-\nabla p+\eta \Delta v$$

where **v** is the flow velocity vector, ρ is the fluid mass density, **f** is the volume density of the body forces acting on the fluid, *p* is the pressure, and η is the dynamic viscosity of the fluid.

The heat transfer model was then simplified by stating the mass conservation hypothesis and by assuming that no slip occurs at the boundaries (see above).

*Heat Transfer*: The heat transfer Equations (2) and (3) were employed as

(2) $$\rho C_{p}u\cdot \nabla T-\nabla \cdot (k\nabla T)\;=\;Q+Q_{p}+Q_{\mathrm{vd}}$$

(3) $$-\nabla \cdot \left(k\nabla T\right)s=Q+Q_{\mathrm{ted}}$$

where *Q* is the general heat source, *Q*_ted_ is the thermoelastic damping, *Q*_vd_ is the viscous dissipation, and *Q*_p_ is the pressure work. In the specific assumptions, *Q*_ted_, *Q*_vd_, and *Q*_p_ are all equal to zero. The following further boundary conditions were applied to the simulation: (1) the system is thermally isolated on the thinner glass side, (2) a boundary heat source with a power density of 510 W m^−2^ has been set on the thin glass side, (3) heat exchange between the environment and the system occurs on the thicker glass side with a constant environmental temperature of 23 °C, (4) at the input, the fluid's temperature is 23 °C, and (5) at the output, no heat is injected from the environment $\left(-\vec{\text{n}}\cdot \vec{\text{q}}\text{=0}\right)$.

*Computational Rendering*: Computational renderings of the appearance of capillary glass in real window systems were generated using the software Rhino v5, SketchUp v14, and V‐Ray v3.2. To make the effect of the fluid as realistic as possible, various features were considered, including refractive index, density, color, and viscosity of the employed glass, water, and an optically neutral filling medium with zero absorption. However, the vast temperature dependence of these material properties is presently not considered. For renderings shown in Figures 2 and 9, the channel size is set to the geometric values of the prototype device (see Figure 10 and Table 1) and the refractive index of the fluid is water with index 1.32.
